# Multifactor relationships between stand structure and soil and water conservation functions of *Robinia pseudoacacia* L. in the Loess Region

**DOI:** 10.1371/journal.pone.0219499

**Published:** 2019-07-10

**Authors:** Xi Wei, Wenjun Liang

**Affiliations:** 1 College of Forestry, Shanxi Agricultural University, Taigu, China; 2 Ji County Station, Chinese National Ecosystem Research Network (CNERN), Beijing, China; University of the Chinese Academy of Sciences, CHINA

## Abstract

Ninety-six sample plots were established for a tree census to explore the multifactor relationships between the soil and water conservation functions and the stand structure in a typical black locust (*Robinia pseudoacacia* L.) plantation in the Caijiachuan watershed of the Loess Plateau, Western Shanxi Province, China. Based on the observational and experimental data, a topography-structure-function model was built using a structural equation modeling (SEM) approach. The latent variables were the topographical factors, horizontal structure, vertical structure, soil and water conservation, and sediment reduction. The results indicated that the horizontal structure of the *Robinia pseudoacacia* L. forest was the most obvious latent variable, which was expressed in the path coefficient (pc = 0.85) corresponding to the sediment reduction; the stand density and tree competition index were the major drivers of the structure, with path coefficients of −0.96 and −0.92 and influence coefficients of −0.997 and −0.998. These factors are easily regulated. Among these factors the stand density of the arbor layer is recommended to be kept stable within the range from 1600 to 1700 trees/hm^2^. These relationships showed that reducing the tree competition index and changing the microtopography could effectively enhance the soil and water conservation functions in this ecologically significant loess area.

## Introduction

Soil and water conservation studies have been conducted on the Loess Plateau since 1950, and the ecological environment of this area has gradually recovered and improved as a result of afforestation and vegetation restoration [1,2]. However, during this process, the artificial forests grew slowly and showed premature senescence, and many even died due to excessive density in afforested areas. Subsequently, the soil water became deficient, groundwater levels gradually fell, and other signs of ecological environmental deterioration appeared. Thus, the expected levels of soil and water conservation and the ecological benefits of artificial afforestation are not currently being met.

To examine the causes of these problems, most studies have focused on the structure and functions of the ecosystem of the Loess Plateau as well as other ecologically sensitive areas. For example, research on the relationships between water and forest growth has shown that chronic water stress can reduce forest growth [3]. Moreover, the changes in soil organic carbon stocks have been evaluated by adopting the recommended soil and water conservation practices in the upper Tana River catchment [4]. Other scholars have presented the results of research on several topics related to forest structure, soil moisture [5], soil physical and chemical properties [6], soil erosion, and soil and water conservation. Zhang et al. proposed that vegetation reconstruction was an effective way to reduce runoff and soil erosion and should be the focus of restoring ecosystems in ecologically sensitive regions in loess areas [7]. Sezgin Hacisalihoglu evaluated the effects of Anatolian black pine on soil erosion and soil properties and found that vegetation could reduce soil loss [8]. Lucas-Borja M.E. et al. investigated the effects of stand age and forest structure on microbiological soil properties, enzymatic activities, and nutrient contents [9]. However, research on the multifactor relationships between the stand structure and the soil and water conservation function is lacking.

Structural equation modeling (SEM) has been increasingly used in ecological studies since Grace first utilized it to study the factors that controlled the species density in herbaceous plant communities [10] and the limitations of species diversity on productivity [11]. The SEM approach usually aims to quantify the relationships between multiple factors and then generates strong and distinct links between theoretical and experimental ideas [12]. The approach has recently been employed in a wide range of environmental and ecological studies. For instance, the plant community drivers of carbon storage in boreal forest ecosystems have been revealed [13], the cascading effects of long-term land-use changes on plant traits and ecosystem functions and the factors that affect plant richness in recovering forests have been assessed to explore ecological integrity [14, 15], the direct and indirect associations of plant species richness with landscape conditions and local environmental factors have been investigated, and the conservation strategies have been developed using environmental indicators [16]. All of these studies utilized SEM as an instrument to evaluate these relationships. However, SEM has rarely been applied in the field of stand structure and soil and water conservation functions except to study the relationships between soil characteristics and stand structure in mixed plantations [17].

Traditionally, water resource conservation and erosion reduction were regarded as the main soil and water conservation functions, but these concepts have been extended to include soil water storage capacity and the preservation, storage, recycling, conversion, and acquisition of soil organic matter, nitrogen, phosphorus, and other nutrients [18]. In this paper, the covariance SEM approach is employed to quantify the relationships between the stand structure and soil and water conservation functions of a typical artificial black locust forest on the Loess Plateau, which is considered an inefficient pure plantation. We consider the climate characteristics, topography and geomorphology of the area, the development of the area, and the forest, water, and soil statuses. The stand structure is further subdivided into the horizontal structure and vertical structure. Moreover, the functions are categorized into three types according to the general classification method of the industry and the regional conditions, including water source conservation, soil conservation, and sediment retention and reduction functions. Then, the mechanism and process of the relationship between the stand structure and the soil and water conservation functions can be quantitatively analyzed, and the multiple relationships between the structural and functional factors can be identified. Based on these results, technology for controlling the structural factors of stands that are sensitive to soil and water conservation functions can be proposed to provide useful references for optimizing forestry construction and preventing soil erosion on the Loess Plateau.

## Materials and methods

### Ethics statement

No specific permits were required for the field studies. We confirmed that the site was not privately owned or protected in any way. The field studies did not involve endangered or protected species.

### Site description

The vegetation of the nested Caijiachuan watershed is characterized by artificial shelterbelts of black locust (*Robinia pseudoacacia* L.) with a forest cover rate of up to 72% that covers an area of 38 km^2^. The Caijiachuan region is located in a typical gully area of the Loess Plateau in Ji County, Shanxi Province, China (35°53’–36°21’ N, 110°27’–111°7’ E; elevations 904–1592 m). Recorded meteorological data show that the long-term mean annual air temperature is 10.2°C, the average annual precipitation is 571 mm and has an uneven distribution, and the frost-free period is 172 days. A Haplic Luvisol (soil classification of the Food and Agriculture Organization of the United Nations) soil type predominates, and the soil is mostly alkaline. The average annual potential evapotranspiration (PET) is 1724 mm.

Ninety-six 20 m × 20 m standard plots were established at the plantation to conduct tree censuses during the summer growing season of 2017. The plots were located on various aspects, including shady, semi-shady, sunny, and semi-sunny aspects. The slopes ranged from 15° to 39° and from 900 to 1300 m above sea level (Table 1). Additional details of the Caijiachuan watershed, taking shrubs and herbs as examples, can be found in the published results of Wei Xi et al. (2018) [17].

**Table 1:** Distributions of aspects, slopes, and elevations of the standard plots in the watershed.

Aspect^a^	Sample quantity	Elevation^b^ /m	Sample quantity	Slope^c^ /°	Sample quantity
Shady	12	900–1000	4	≤15	8
Semi-shady	48	1000–1150	44	16–25	48
Sunny	16	1150–1300	48	26–35	36
Semi-sunny	20	>1300	0	≥36	4

^a^The distribution of aspect tends to be heterogeneous, and shady conditions are more common than sunny conditions, which is in consistent the plant distribution.

^b^In the study region, the low-elevation (900–1000 m) areas are mainly agricultural lands or areas where people live, so very few low-elevation sites are available for afforestation; the high-elevation (>1300 m) areas are mainly covered with natural forests, so there are no high-elevation plantations; the mid-elevation areas (1000–1300 m) are the main afforestation areas of *Robinia pseudoacacia* L plantations.

^c^The lands featuring gentle slopes (≤15°) are usually cropland, and those that are dangerously steep (≥36°) are difficult to access. As such, very few of these types of sites are available for afforestation, whereas many sites with steep slopes (16°–35°) are commonly used for afforestation in China to restore vegetation and improve the environment.

### Field surveys and data acquisition

Tree censuses were conducted for all stems with diameters at breast height (DBHs)≥5 cm. In all of the investigated even-aged stands in these plots, the DBH, tree height, and tree crown area were measured, and the leaf area indices (LAIs) of the quadrats were collected using a LAI-2000 (LI-COR Company, Lincoln, NE, USA) vegetation canopy analyzer. The canopy density, stand density, uniform angle [19], neighborhood comparison [20], tree competition index [21], and stand layer index [22] were calculated.

According to the trophic classification scheme for soil and water conservation functions [23], indicators of water resource conservation and soil protection were confirmed. Three replicated samples were established per plot, and mixed soil samples (0–60 cm) were collected using the cutting ring method to represent the soils of *Robinia pseudoacacia* L. forests in this area. The soil moisture content was determined using the drying approach, and the maximum water holding capacity (WHC) was measured using the soil infiltration method. After the air-dried soil was sieved (0.15 mm sieve), the soil organic matter (SOM), total nitrogen (TN), total phosphorus (TP), ammonia-nitrogen (NH_3_-N), nitrate-nitrogen (NO_3_-N), and available phosphorus (AP) contents were measured indoors with a SmartChem-200 (AMS/Alliance Instruments, Paris, France) discrete wet chemistry analyzer [24,25]. The infiltration properties of the slope were studied using the double loop infiltration approach. Canopy interception was indirectly calculated by measuring the rain outside and inside the forest using a tipping bucket self-metering rain gauge. The average runoff yield and average sediment yield were obtained by observing the runoff plot in each standard plot in the forest. The major geographical and biological characteristics of the investigated plots are summarized in Table 2.

**Table 2:** Characteristics of the *Robinia pseudoacacia* L. plantations in the watershed.

Stands and Soil Characteristics	Minimum	Maximum	Average
Slope (°)	15	39	25
Elevation (m)	990	1220	1147
DBH^a^ (cm)	5.0	30.4	9.9
Tree height (m)	1.7	20.2	8.6
Crown area (m^2^)	0.2	52.5	8.0
Canopy density	0.38	0.87	0.62
Stand density (trees∙hectare^−1^)	500	3500	1746
LAI^b^	0.88	4.50	2.06
Uniform angle	0.25	0.94	0.55
Neighborhood comparison	0.125	0.75	0.50
Tree competition index	1.08	3.77	2.11
Stand layer index	0	0.48	0.32
Soil moisture content (%)	5.66	33.97	12.93
Maximum WHC^c^ (%)	34.60	75.45	48.20
SOM^d^ (g∙kg^−1^)	1.31	55.60	12.63
TN^e^ (g∙kg^−1^)	0.13	2.22	0.66
TP^f^ (g∙kg^−1^)	0.03	7.60	0.68
NH_3_-N ^g^ (mg∙kg^−1^)	2.79	42.07	18.41
NO_3_-N^h^ (mg∙kg^−1^)	0.12	88.40	11.05
AP^i^ (mg∙kg^−1^)	0.16	117.65	33.50
Canopy interception rate (%)	8.98	26.87	18.62
Water retention rate of litter in the undecomposed layer (%)	2.95	10.27	4.68
Water retention rate of litter in the semidecomposed layer (%)	2.06	5.94	4.05
Soil infiltration rate (mm/h)	79.41	616.86	325.75
Average runoff yield (mm)	32.11	72.16	50.76
Average sediment yield (t·km^-2^)	271	826	413

^a^ DBH, diameter at breast height.

^b^ LAI, leaf area index.

^c^WHC, water holding capacity.

^d^SOM, soil organic matter.

^e^TN, total nitrogen.

^f^TP, total phosphorus.

^g^NH_3_-N, ammonia-nitrogen.

^h^NO_3_-N, nitrate-nitrogen.

^i^AP, available phosphorus.

### Structural equation modeling

Models constructed using SEM are multivariate statistical models [26] that are applied to express the relationships among many different indicators of an ecosystem, including path, causal, and direct versus indirect analyses [27,28]. These models have advantages over regression models because they construct all hypothetical causal links between the predictor variables and the dependent variables [29–31].

Based on previous knowledge of the ecological system [31] and statistical data obtained from field surveys, SEM methods can be used to evaluate the structure and function of an ecosystem [32], mainly the stand structure and the soil and water conservation functions in this study. Because the climate, hydrology, and other environmental conditions in the area were mostly consistent, we considered the topographical factors (*ξ*_1_), horizontal structure (*ξ*_2_), and vertical structure (*ξ*_3_) as potential exogenous variables. In our hypothesis, the function was the focus; therefore, soil and water conservation (*η*_1_) and sediment reduction (*η*_2_) were taken as potential endogenous variables. Furthermore, SEM in ecology is closely related to a combination of several statistical approaches to model multivariate relationships [33,34], and classical SEMs are composed of two measurement equations and a structural equation [cause-effect] [35–37]. The measurement models described the respective relationships between the latent and observed variables, and the structural model expressed the relationships between the five potential variables.

Because the sample is subject to random selection in the survey, the slope (*x*_1_), aspect (*x*_2_), elevation (*x*_3_), DBH (*x*_4_), tree crown area (*x*_5_), stand density (*x*_6_), canopy density (*x*_7_), uniform angle (*x*_8_), neighborhood comparison (*x*_9_), tree competition index (*x*_10_), tree height (*x*_11_), LAI (*x*_12_), and stand layer index (*x*_13_) were determined to be the indices of the hypothesis model belonging to the exogenous observation variables. The corresponding functional indices, such as the canopy interception rate (*y*_1_), water retention rate of litter in the undecomposed layer (*y*_2_), water retention rate of litter in the semidecomposed layer (*y*_3_), soil infiltration rate (*y*_4_), soil moisture content (*y*_5_), maximum WHC (*y*_6_), SOM (*y*_7_), TP (*y*_8_), TN (*y*_9_), NH_3_-N (*y*_10_), NO_3_-N (*y*_11_), AP (*y*_12_), average runoff yield (*y*_13_), and average sediment yield (*y*_14_), were determined as the endogenous observation variables. In addition, the residuals of the five latent variables and twenty-six observed variables were considered during the modeling process.

The initial model reflected the relationships among the topography, stand structure, and soil and water conservation functions with the aid of a path diagram based on previous knowledge and a preliminary qualitative study. In the model, the path coefficients were calculated using the maximum likelihood (ML) approach [34], and the coefficients represented the extents of their relationships. A chi-square value (*χ*^2^) and the respective p-values associated with it were used to examine whether the fit between the model and data was satisfactory [27,38]. The *χ*^2^, degrees of freedom (*df*, 0 ≤ *χ*^2^/*df* ≤ 3), probability level (*p* > 0.05), root mean square error of approximation (RMSEA) (0 ≤ RMSEA ≤ 0.05) [37], goodness-of-fit index (GFI) (0.9 ≤ GFI ≤ 1.00), and comparative fit index (CFI) (0.9 ≤ CFI ≤ 1.00) were regarded as the “best” parameter ranges, representing the highest compliance with the model, and a CFI ranging from 0.7 to 0.90 was considered “tolerable” [39].

If these parameters of the initial model were not within the proper range, the model was corrected according to the characteristics of the measurable variables that were investigated using the “modification index” and “critical ratio (CR) for difference” methods provided by the Amos 22.0 software (IBM/International Business Machines Corporation, Armonk, USA) package. The former method was primarily used in this study.

## Results

### Feature, reliability and validity analyses

Before the SEM path diagram of *Robinia pseudoacacia* L. plantations was created, their features were analyzed. Only the characteristic indicators that passed the reliability and validity tests were used to construct the model.

**Fig 1:**
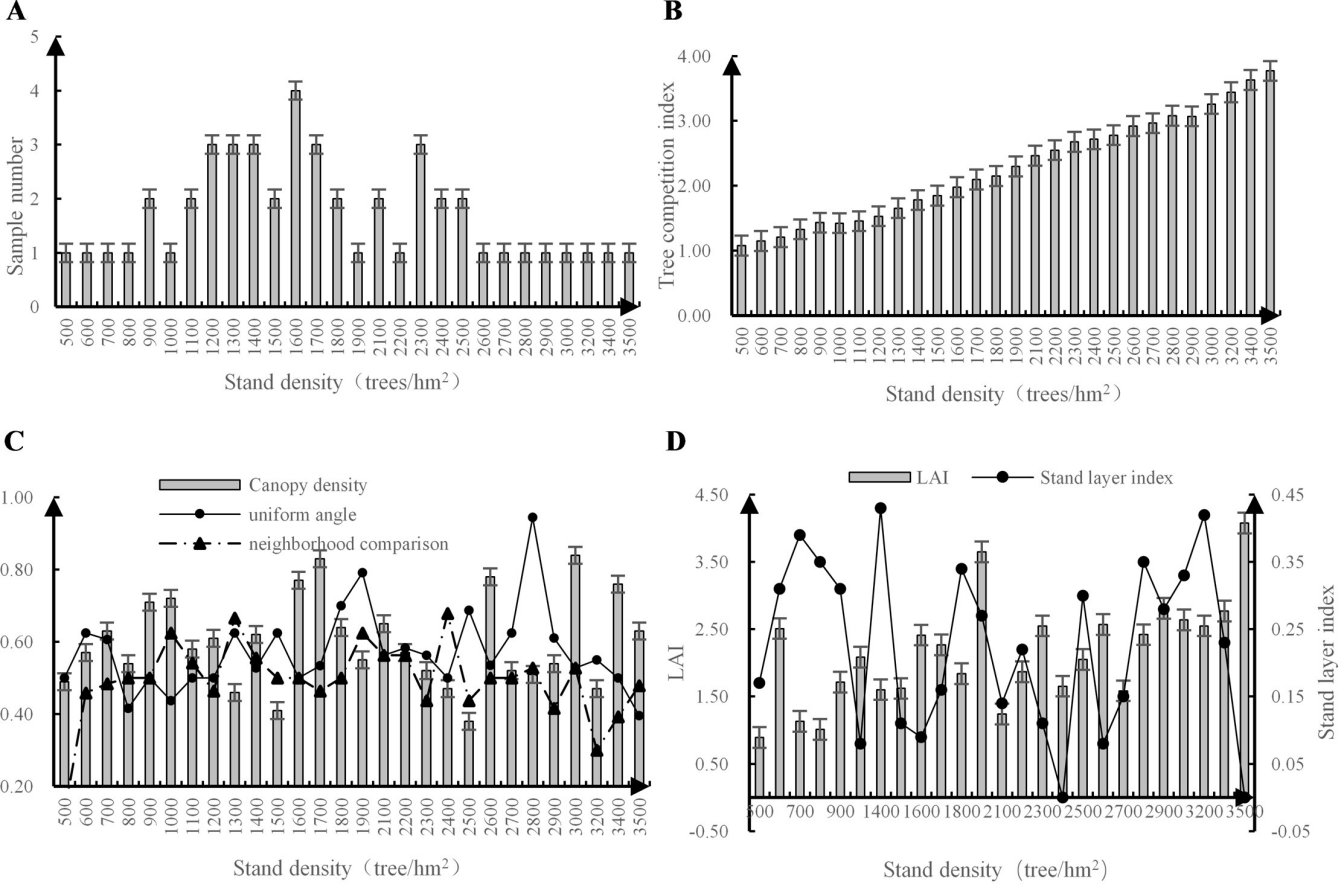
The main stand structure characteristics of *Robinia pseudoacacia* L. (A) Stand density. (B) Tree competition index. (C) Canopy density, uniform angle, and neighborhood comparison. (D) LAI and stand layer index.

Among the random plots, the density of the *Robinia pseudoacacia* L. stands ranged from 500 to 3500 trees/hm^2^ (Fig 1A). The competition index ranged from 1.08 to 3.77, gradually increasing with increasing stand density (Fig 1B). The canopy density ranged from 0.38 to 0.87, with peaks at 1600, 1700, and 3000 trees/hm^2^. The overall trends of the uniform angle and neighborhood comparison, which increased with stand density, were similar. Therefore, the distribution was mostly cluster-like, and the differences in the stand sizes were obvious (Fig 1C). The LAI ranged from 0.88 to 4.50; the overall trend of the LAI also gradually increased with increasing stand density, but the peak values were reached at densities of 600, 1900, and 3500 trees/hm^2^. The stand layer index ranged from 0 to 0.48, and the forest stratification was mostly concentrated in 1–2 layers, peaking at 1400 and 3200 trees/hm^2^ (Fig 1D). The data indicate that the stand structure indices between the stand densities of 1600 and 1700 trees/hm^2^ were moderate.

**Fig 2:**
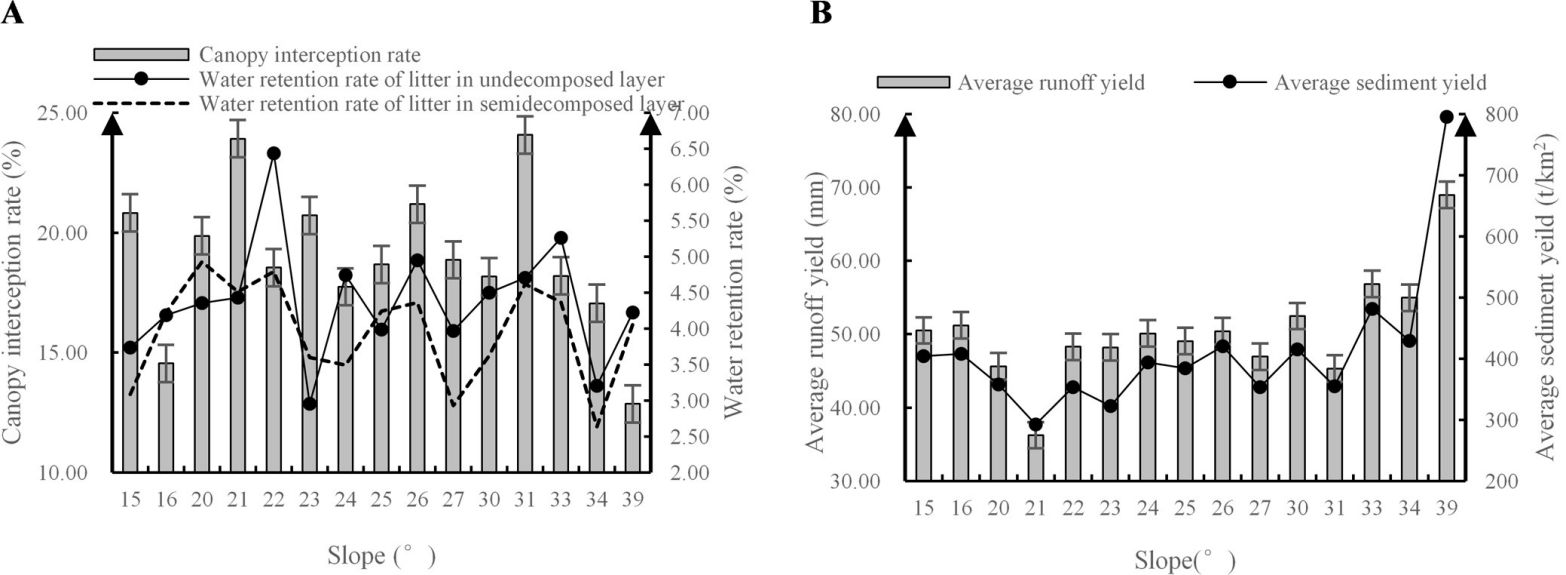
The soil and water conservation function characteristics of *Robinia pseudoacacia* L. (A) Canopy interception and water retention of the litter. (B) Average runoff and sediment yields.

As shown in Fig 2, the average canopy interception rate of *Robinia pseudoacacia* L. exhibited a double-peak fluctuation curve with peaks at 21° and 31°. The WHC of the litter in the undecomposed layer was generally stronger than that in the semidecomposed layer. The overall trends of the average runoff and sediment yield indicators increased gradually with increasing slope. The peak runoff and sediment values were 68.98 mm and 795 t/km^2^, respectively, which occurred on the steep slope of 39°.

After the feature analysis, these indicators must be inspected for reliability (using Cronbach’s alpha coefficient (α≥0.9)) and validity (using the KMO metrics (KMO≥0.8) and Bartlett spherical tests (sig.< 1%)). The exploratory analysis was conducted in the SPSS 19.0 software (IBM/International Business Machines Corporation, Armonk, USA). The results showed that the slope, aspect, elevation, DBH, tree crown area, canopy density, stand density, uniform angle, neighborhood comparison, tree competition index, tree height, LAI, stand layer index, canopy interception rate, water retention rate of litter in the undecomposed layer, water retention rate of litter in the semidecomposed layer, soil infiltration rate, soil moisture content, maximum WHC, SOM, TP, average runoff yield, and average sediment yield met the inclusion criteria, and these indicators were incorporated into the model.

### Model construction and correction

The dataset was used to build an SEM model that conformed to a multivariate normal distribution using the Amos 22.0 software (IBM/International Business Machines Corporation, Armonk, USA). ML estimation was used to quantitatively analyze the potential and observed variables. Then, based on theoretical assumptions and generally well-known previous experience, the path diagram, parameters, and path coefficients of the model could be obtained after the initial model run.

**Fig 3:**
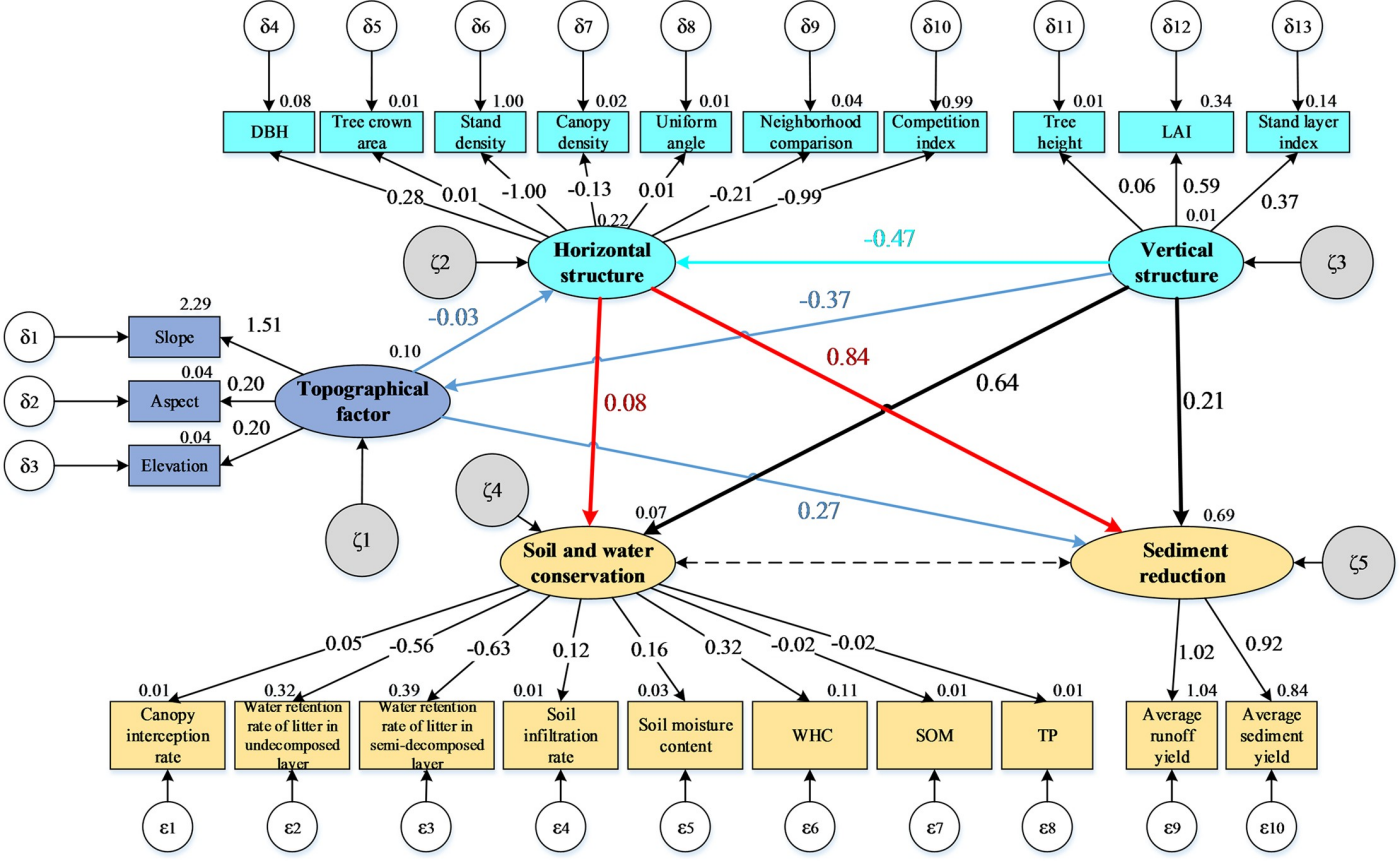
The initial structural equation model of the coupled relationships between the stand structure and the soil and water conservation functions of *Robinia pseudoacacia* L. Notes: ^a^The hypothesized initial model used to predict the topographical factors, horizontal structure, vertical structure, soil and water conservation, and sediment reduction is based on soil and water conservation science and general experience. ^b^A rectangular box is used for each observed variable, including the measurement errors, and the numbers on the single arrows correspond to the standardized path coefficients of the initial model. Values outside the rectangular boxes are the means of the indicators, and values outside the ovals are the residual errors before modification. ^c^In Figs 3 and 4, DBH is the acronym for diameter at breast height; LAI is the abbreviation for leaf area index; WHC is the acronym for soil maximum water holding capacity; SOM is the abbreviation for soil organic matter; and TP is the acronym for total phosphorus.

In the initial model, the chi-square (*χ*^2^) test statistic value was 78.756 with 18 *df*, and the model had a significant probability (*p*) value of 0.0001 (<0.05) (Fig 3), leading to the rejection of the null hypothesis. In addition, the RMSEA was 0.189 (greater than 0.05), under the null hypothesis of a “close fit”, and the GFI, normative fit index (NFI), incremental fit index (IFI) and CFI values were all less than 0.7. Thus, the adaptability of the hypothetical model to the observed data had to be corrected (Table 3).

**Table 3:** Fitting parameters describing the coupled relationship between the stand structure and the soil and water conservation functions of *Robinia pseudoacacia* L. in the watershed.

Index Name	Evaluation Criterion	Initial Model	Corrected Model
Ratio of chi-square to freedom (*χ*^2^/*df*)	1~3. When the ratio is less than 1, the model is over-adapted; when it is between 1 and 3, the model is well-adapted; and when it is greater than 3, the model is poorly fitted.	4.389	1.715
Significant probability (*p*)	>0.05.	0.0001	0.068
Goodness-of-fit index (GFI)	0~1. Values greater than 0.7 are tolerable, and values closer to 1 are better.	0.632	0.902
Normative fit index (NFI)	0~1. Values greater than 0.7 are tolerable, and values closer to 1 are better.	0.468	0.826
Incremental fit index (IFI)	0~1. Values greater than 0.7 are tolerable, and values closer to 1 are better.	0.533	0.841
Comparative fit index (CFI)	0~1. Values greater than 0.7 are tolerable, and values closer to 1 are better.	0.524	0.832
Root meant square error of approximation (RMSEA)	<0.05. Smaller values are better.	0.189	0.039
Akaike information criterion (AIC)	Smaller values are better.	598.000	266.726
Bayes criterion (BCC)	Smaller values are better.	600.141	223.515

**Fig 4:**
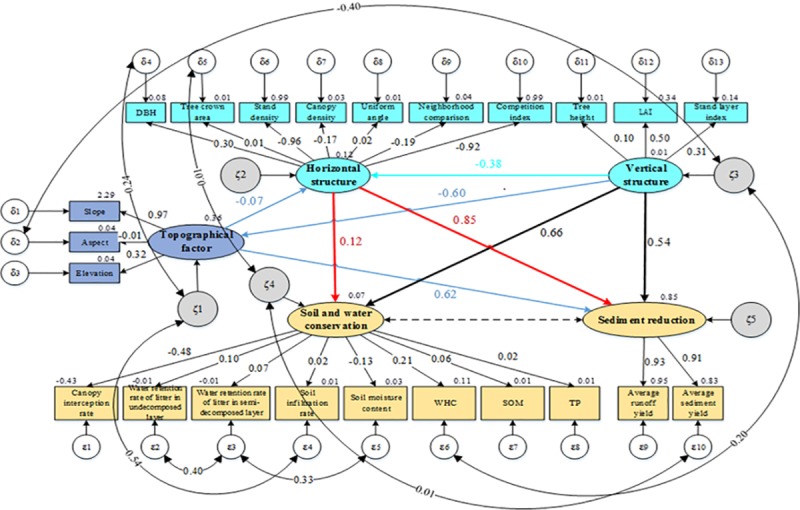
The modified structural equation model of the coupled relationship between the stand structure and the soil and water conservation functions of *Robinia pseudoacacia* L. Notes: The numbers on the single arrows correspond to the standardized path coefficients, and those on the double arrows correspond to the correlation coefficients. Values outside the rectangular boxes are the means of the indicators, and values outside the ovals are the residual errors after modification.

“Modification Indices” hints from the Amos software were selected to modify the initial model because the method and data conformed to current theoretical research and qualitative analyses of the conclusions. Double arrows between the residuals of each variable were added to correlate the observed variables. After the addition of several arrows and repeated adjustments, the parameters in Table 3 were acceptable, and the modified model exhibited a good fit, supporting the null hypothesis (Fig 4). The *χ*^2^-test (*χ*^2^/*df* = 1. 715, *p* = 0.068 (>0.05)), RMSEA = 0.039 (<0.05), and fit indices (>0.8) verified that the model and the data had high adaptability (Table 3). The inspection of the other fitness indicators indicated that they also matched the standards, indicating that the fit of the model had improved. Therefore, the conclusions from the model were feasible and realistic.

### Model explanation

#### Coupling mechanisms between latent variables

The topographical factor had negative effects on the horizontal structure and vertical structure and a positive effect on sediment reduction (Fig 4), with path coefficients of −0.07, −0.60, and 0.62, respectively. Numerically, the effects of topography on the vertical structure and sediment reduction were far greater than the impacts on the horizontal structure. The horizontal structure had a negative impact on the vertical structure, with a path coefficient of −0.38, and it had positive impacts on soil and water conservation and sediment reduction, with path coefficients of 0.12 and 0.85, respectively. The vertical structure had positive impacts on soil and water conservation and sediment reduction, with path coefficients of 0.66 and 0.54, respectively. Standardized influence coefficients characterized the effects of the latent variables, which were calculated using the SEM methods (Table 4). The influence coefficients of the topography on the horizontal and vertical structures were −0.073 and −0.598, respectively, and both of these impacts were direct; the total impact coefficient of the topography on the sediment reduction was 0.557, with a direct impact of 0.619 and an indirect impact of −0.062. The influence coefficients of the horizontal structure on sediment reduction and soil and water conservation were 0.851 and 0.066, respectively, and both were direct impacts. The influence coefficient of the horizontal structure on the vertical structure was −0.336, with a direct impact of −0.379 and an indirect impact of 0.044. The total, direct, and indirect impact coefficients of the vertical structure on sediment reduction were −0.111, 0.545, and −0.656, respectively, and the total, direct, and indirect impact coefficients of the vertical structure on soil and water conservation were 0.012, −0.022, and 0.035, respectively. The relationship between sediment reduction and soil and water conservation was a correlation but not a causality.

**Table 4:** Influence coefficients of latent variable standardization in the model.

Effect latent variables	Standardized total impact	Standardized direct impact	Standardized indirect impact
Horizontal structure and topography	−0.073	−0.073	0^a^
Vertical structure and topography	−0.598	−0.598	0^a^
Sediment reduction and topography	0.557	0.557	−0.062
Vertical structure and horizontal structure	−0.336	−0.379	0.044
Sediment reduction and horizontal structure	0.851	0.851	0^a^
Soil and water conservation horizontal structure	0.066	0.066	0^a^
Sediment reduction and vertical structure	−0.111	0.545	−0.656
Soil and water conservation and vertical structure	0.012	−0.022	0.035

^a^The influence coefficients between the other latent variables were zero.

The coupling mechanisms and relationships among these factors could be explained by analyzing the data presented above. First, more adverse effects on the horizontal structure occurred when the topography was more variable, and the impacts on the vertical structure were especially pronounced. In addition, the sediment-reducing function decreased as the sediment reduction value increased. Second, the horizontal and vertical structures had internal constraints on each other. Moreover, the horizontal structure exerted a large positive effect on sediment reduction and a small positive effect on soil and water conservation. Third, the numerical value of the vertical structure negatively impacted the sediment reduction and positively impacted the soil and water conservation. Furthermore, microtopography and stand structure should be optimized to improve soil and water conservation. In practice, comprehensive consideration should be given to the topographic factors and the horizontal versus vertical structures, which should be adjusted accurately in a directional and quantitative manner to promote water conservation, soil fertility, and sediment retention. However, the topography and stand structure adjustments should be moderate to avoid accumulated negative effects.

#### Relationships between latent and observed variables

The extent and effect of the influence between the latent variables and observed variables were also reflected in the path coefficients of the fitted model (Fig 4). In the diagram, the topography influenced the slope and elevation positively and significantly, with path coefficients of 0.97 and 0.32, respectively. Among the observed variables of the horizontal structure, DBH, tree crown area, and uniform angle showed positive effects, while the others showed negative effects, with the stand density and tree competition index exhibiting large effects. All three observed variables that affected the vertical structure showed positive effects, and the influence of LAI was a dominant factor. For soil and water conservation, the maximum WHC, soil infiltration rate, SOM, and TP had positive effects, while the remaining factors had negative effects. The path coefficient of the canopy interception rate was much greater than those of the other factors. The average runoff yield and average sediment yield both had positive effects on sediment reduction, and the runoff yield had a slightly greater effect than the sediment yield.

The standardized influence coefficients also characterized the effect of each latent variable on each observed variable (Table 5). As an exogenous latent variable, topography had positive effects on some observed variables, including the runoff yield, sediment yield, and slope, and it had negative effects on other observed variables. The largest effect was on the slope, with a direct impact coefficient of 0.97, the effects of topography on the elevation, runoff yield, and sediment yield were also significant, while the effects on the other factors were less significant and even close to zero. The horizontal structure had positive effects on the runoff yield and sediment yield. In contrast, the horizontal structure had negative effects on the canopy interception rate, stand density, and tree competition index, etc. The effects of the horizontal structure on the stand density, tree competition index, runoff yield, sediment yield, and canopy interception rate were greater than the effects on the other observed variables, with influence coefficients of −0.997, −0.998, 0.874, 0.774, and −0.836, respectively. In addition, the horizontal structure had a less significant impact on the water retention rate of the litter in the semidecomposed layer, soil moisture content, DBH, and neighborhood comparison than on the other factors. The observed variables of the horizontal structure were all direct effects. Furthermore, the latent variable of the vertical structure positively impacted the LAI, stand density, and tree competition index, and it negatively influenced the sediment yield, elevation, and slope. Among these factors, the vertical structure had more significant effects on the elevation, slope, LAI, stand layer index, stand density, and tree competition index than the other factors; the effects of the tree height, LAI, and stand layer index were direct, but the effects of the other factors were indirect.

**Table 5:** Influence coefficients of the observed variables in the structure equation model of *Robinia pseudoacacia* L.

Observed Variables	Standardized total impact					Standardized direct impact					Standardized indirect impact				
	Topography	Horizontal structure	Vertical structure	Sediment reduction	Soil and water conservation	Topography	Horizontal structure	Vertical structure	Sediment reduction	Soil and water conservation	Topography	Horizontal structure	Vertical structure	Sediment reduction	Soil and water conservation
TP	0.003	-0.04	0.005	0	0	0	0	0	0	0	0.003	-0.04	0.005	0	0
SOM	0.001	-0.011	0.001	0	0	0	0	0	0	0	0.001	-0.011	0.001	0	0
Runoff yield	**0.506**^a^	**0.774**^a^	-0.101	**0.909**^a^	0	0	0	0	**0.909**^a^	0	**0.506**^a^	**0.774**^a^	-0.101	0	0
Sediment yield	**0.571**^a^	**0.874**^a^	-0.114	**0.976**^a^	0	0	0	0	**0.976**^a^	0	**0.571**^a^	**0.874**^a^	-0.114	0	0
Canopy interception rate	0.061	**-0.836**^a^	0.1	0	0	0	0	0	0	0	0.061	**-0.836**^a^	0.1	0	0
Water retention rate of litter in the undecomposed layer	-0.007	0.095	-0.011	0	0	0	0	0	0	0	-0.007	0.095	-0.011	0	0
Water retention rate of litter in the semidecomposed layer	-0.008	0.113	-0.014	0	0	0	0	0	0	0	-0.008	0.113	-0.014	0	0
Soil infiltration rate	-0.001	0.01	-0.001	0	0	0	0	0	0	0	-0.001	0.01	-0.001	0	0
Soil moisture content	-0.014	0.188	-0.022	0	0	0	0	0	0	0	-0.014	0.188	-0.022	0	0
WHC	0.004	-0.052	0.006	0	0.001	0	0	0	0	0.001	0.004	-0.052	0.006	0	0
Elevation	0.316	0	-0.189	0	0	0.316	0	0	0	0	0	0	-0.189	0	0
Aspect	-0.005	0	0.003	0	0	-0.005	0	0	0	0	0	0	0.003	0	0
Slope	**0.97**^a^	0	**-0.64**^a^	0	0	**0.97**^a^	0	0	0	0	0	0	**-0.64**^a^	0	0
LAI	0	0	0.496	0	0	0	0	0.496	0	0	0	0	0	0	0
Tree height	0	0	0.102	0	0.002	0	0	0.102	0	0	0	0	0	0	0.002
Stand layer index	0	0	0.311	0	0	0	0	0.311	0	0	0	0	0	0	0
Crown area	0	0.001	0	0	0	0	0.001	0	0	0	0	0	0	0	0
DBH	-0.022	0.302	-0.101	0	0	0	0.302	0	0	0	-0.022	0	-0.101	0	0
Canopy density	0.012	-0.167	0.056	0	0	0	-0.167	0	0	0	0.012	0	0.056	0	0
Stand density	0.073	**-0.997**^a^	0.334	0	-0.004	0	**-0.997**^a^	0	0	0	0.073	0	0.334	0	-0.004
Tree competition index	0.073	**-0.998**^a^	0.335	0	0	0	**-0.998**^a^	0	0	0	0.073	0	0.335	0	0
Neighborhood comparison	0.014	-0.194	0.065	0	0	0	-0.194	0	0	0	0.014	0	0.065	0	0
Uniform angle	-0.001	0.019	-0.006	0	0	0	0.019	0	0	0	-0.001	0	-0.006	0	0

^a^The data with absolute values greater than 0.5 are shown in bold to facilitate the identification of factors with significant total, direct and indirect influences among the latent and observed variables.

As an endogenous latent variable, sediment reduction had direct positive impacts on the runoff yield and sediment yield, with influence coefficients of 0.909 and 0.976, respectively. In contrast, soil and water conservation had a direct positive impacts on the WHC, an indirect positive impacts on the tree height, and an indirect negative impacts on the LAI.

## Discussion

### Topography directly impacts horizontal structure

The aspect, slope, slope length, slope position, and elevation are generally considered topographical factors, and they impact the horizontal and vertical structures, including the DBH, tree height, stand density, and LAI, as well as their dynamics. Significant interactions or remarkable dissimilarities existed between the influences of aspect and elevation on the stand structure [40], including the tree height [41] and other factors. Thus, the forest should be adapted to the different temperatures and other conditions at various elevations [42].

The subdivision of the forest structure into horizontal and vertical structures further reflected the different effects of the terrain on the spatial stand structure. When the dataset was subjected to the corrected model, the topography exhibited close relationships with the horizontal and vertical structures; their path coefficients were −0.07 and −0.60, respectively. Thus, topography could have not only a direct effect on soil and water conservation but also an indirect effect via the stand structure. Changing the topography (e.g., increasing the slope) should negatively and indirectly affect the relationships between the stand structure and the soil and water functions.

### Horizontal and vertical structures impact each other

Previous studies have frequently demonstrated the obvious relationships between the horizontal structure and vertical structure; however, these relationships have rarely been quantified. The DBH and tree height, representing the two dimensions, were considered complex interactions [43]; thus, the horizontal and vertical vegetation structures could be efficiently assessed to inform future studies of biodiversity and ecosystem services [44].

The horizontal and vertical structures were regarded as two dimensions of the stand structure of *Robinia pseudoacacia* L., and there were negative interactions between these structures. The path coefficient was −0.38, and the total effect was −0.336. The vertical structure had significant effects on the stand density and tree competition index, with influence coefficients of 0.334 and 0.335, respectively. This result indicated that when the stand structure affected the function, there was a competitive relationship between forest growth and stand structure in both the horizontal and vertical directions. The positive variations in the horizontal structure could limit the improvements to the vertical structure, resulting in negative vertical variations.

### Horizontal structure impacts functional factors as a significant structural factor

Previous studies revealed some of the principles of the interaction between forest structure and function. Soil variables were observed to differ in terms of soil TN across different canopy types [45], and the forest structure was found to influence microbiological soil properties [46]. However, most studies have focused on a single factor. The novel approach that was applied in this study revealed that the horizontal structure had the most significant negative effects on the stand density and tree competition index, with path coefficients of −0.96 and −0.92 and influence coefficients of −0.997 and −0.998, respectively. This result illustrated that among the seven factors, the stand density and tree competition index dominated the horizontal structure, impacting the soil and water conservation functions. The feature and related analysis results indicated that there was a close positive relationship between the observed stand density and the tree competition index variables, and both variables were significantly related to the DBH, stand layer index, and LAI at the 0.05 level. Thus, there was obvious intraspecific competition among trees in *Robinia pseudoacacia* L. forests. The DBH, stand density, and tree competition index comprehensively controlled the horizontal structure.

Additionally, the average canopy interception rate of the *Robinia pseudoacacia* L. forest exhibited a double-peaked fluctuation curve, and the feature analysis indicated that the average canopy interception rate was significantly negatively correlated with the runoff yield and sediment yield. Based on these results, the dataset was mined deeply using SEM methods. The path coefficients of the horizontal structure with soil and water conservation and sediment reduction were 0.12 and 0.85, respectively. The horizontal structure had the most significant indirect effects on the runoff yield, sediment yield, and canopy interception rate, with influence coefficients of 0.874, 0.774, and −0.836, respectively, and it directly impacted soil and water conservation and sediment reduction. Canopy interception increased after the horizontal structure was optimized (mainly by decreasing the stand density and tree competition or increasing other horizontal structural indices). Moreover, the runoff yield and sediment yield under forests should be decreased to promote sediment reduction and soil and water conservation. The improving effect of the former was more prominent than that of the latter. Therefore, the multifactor result of the model was generally consistent with the two-factor correlation analysis, but it highlighted the coupled process and mechanism of the impact of the horizontal structure on soil and water conservation and sediment reduction functions.

### Vertical structure also impacts functional factors

The functions of canopy composition and stratification, which are commonly considered typical vertical structural indicators, have been widely studied [47,48], in contrast to the soil and water conservation functions. In the results of this study, the effects of the three observed factors on the vertical structure were all obvious, and the LAI and stand layer index had greater impacts than the tree height.

Combined with the correlation results and model analysis, the relationships between two factors, such as the canopy interception rate and soil moisture content, maximum WHC and water retention rate of litter in the undecomposed layer, ammonia-nitrogen and sediment yield, and ammonia-nitrogen and nitrate-nitrogen, were significantly correlated. Moreover, the SEM results showed that the path coefficients between the vertical structure and the soil and water conservation and sediment reduction were 0.66 and 0.54, respectively. The coefficients of the effects of the vertical structure on the observed variables ranged from 0.001 to 0.200. The indirect effect of the vertical structure was weaker than that of the other latent variables. However, these variables could not be ignored due to their large path coefficients. If the vertical structure was optimized (e.g., increasing tree height, LAI, and stand layer index), various soil and water conservation functions would be enhanced to varying degrees. Therefore, the optimization of sediment reduction had a stronger impact than the optimization of soil and water conservation.

### Configuration of the stand structure optimization measures

The final objective of this study was to develop more accurate optimization measures of the stand structure to improve the soil and water conservation functions, which is an improvement over previous studies [17]. Many effective approaches have been adopted to optimize the stand structure, such as thinning [49], tending management [50,51], optimizing the stand structure [52], forestry programming [53] and altering the microtopography [54]. The results of this study, which combined multiple methods of analyzing features and building models, indicated that the stand density, tree competition index, LAI, and stand layer index were significant and sensitive for regulatory measures to enhance the soil and water conservation functions of *Robinia pseudoacacia* L. plantations. These factors should be adjusted by adopting appropriate forest management techniques, such as thinning, cutting, replanting, irrigation, and fertilization.

Over the next decade, the stand structure will be continuously optimized using moderate management, and the structure should gradually stabilize. The stand density and canopy density of the arbor layer will stabilize within the ranges from 1600 to 1700 trees/hm^2^ and from 0.6 to 0.7, respectively. Simultaneously, sapling renewal will become stable, the diversity of shrubs and grasses will increase, the forest gradients will remain clear, and the litter under the forest will be rich and easily decomposed. If the forest grows well after long-term management, the horizontal and vertical structures will become more appropriate, and the entire forest will become healthier. Under these conditions, the soil and water conservation functions of the *Robinia pseudoacacia* L. forest can achieve optimal conditions.

## Conclusions

This paper showed the comprehensive coupled process and relationships between the stand structure and the soil and water conservation functions using SEM methods. The horizontal structure of a *Robinia pseudoacacia* L. forest was an obvious latent variable. The effects of the horizontal structure on the sediment reduction and water conservation functions were stronger than those of the other variables, especially the vertical structure of the stand space. The results of this study were generally consistent with the results of previous research [55,56] and were more quantitative. The structural factors that significantly influenced the soil and water conservation functions were the stand density, tree competition index, and LAI, and the sensitive functional factors that were significantly affected by the structural factors were mainly the runoff yield, sediment yield, and canopy interception rate. After the various effects of the soil and water conservation functions were superimposed, outstanding performance was related to sediment reduction and water conservation. In addition, topographical factors played significant roles in strengthening or weakening the coupled process.

Therefore, the regulation of stand structure that aims to promote various types of soil and water conservation functions should be directly or indirectly implemented in response to the sensitive indicators described above, focusing more on functionality than before. To achieve the stand structure of typical forests on the Loess Plateau that is appropriate for soil and water conservation (optimized regulation target), quantitative recommendations for structural optimization are proposed. For instance, we could increase or decrease the stand density to the range of 1600 to 1700 trees/hm^2^, implement low-efficiency forest transformation technology, reduce tree competition, and reduce the slope of the microtopography. After implementing such approaches, the previous low-efficiency pure *Robinia pseudoacacia* L. forests on the Loess Plateau will be transformed into heterogeneous mixed forests with the multiobjective optimization of strong soil and water conservation functions.

## Supporting information

S1 File The basic information of the samples. (PDF)
