# Supplementary material for: Multifactor relationships between stand structure and soil and water conservation functions of Robinia pseudoacacia L. in the Loess Region
Source: PLoS One. 2019 Jul 10;14(7):e0219499. doi: 10.1371/journal.pone.0219499 (PMC6619771; doi:10.1371/journal.pone.0219499)
Supplement: S1 File — (PDF) [file pone.0219499.s001.pdf]

Sequence number	Sample number	Slope (°)	Altitude (m)	Stand density (trees·hectare^{-1})
1	Sample 1	15	1100	1200
2	Sample 2	15	1100	2800
3	Sample 3	15	1100	1700
4	Sample 4	15	1100	1200
5	Sample 5	25	1120	1600
6	Sample 6	25	1120	2200
7	Sample 7	25	1120	2800
8	Sample 8	25	1120	1700
9	Sample 9	20	1130	1700
10	Sample 10	20	1130	2300
11	Sample 11	20	1130	1700
12	Sample 12	20	1130	1300
13	Sample 13	15	1120	2500
14	Sample 14	15	1120	1600
15	Sample 15	15	1120	3400
16	Sample 16	15	1120	2600
17	Sample 17	26	1140	1300
18	Sample 18	26	1140	700
19	Sample 19	26	1140	500
20	Sample 20	26	1140	2300
21	Sample 21	23	1180	2500
22	Sample 22	23	1180	2300
23	Sample 23	23	1180	2900
24	Sample 24	23	1180	1900
25	Sample 25	30	1210	1400
26	Sample 26	30	1210	2100
27	Sample 27	30	1210	1400
28	Sample 28	30	1210	800
29	Sample 29	34	1220	1700
30	Sample 30	34	1220	1600
31	Sample 31	34	1220	2400
32	Sample 32	34	1220	1800
33	Sample 33	31	1190	3200
34	Sample 34	31	1190	2300
35	Sample 35	31	1190	3000
36	Sample 36	31	1190	2300
37	Sample 37	33	1200	1800
38	Sample 38	33	1200	1600
39	Sample 39	33	1200	1700
40	Sample 40	33	1200	2100
41	Sample 41	22	1120	2300
42	Sample 42	22	1120	1900
43	Sample 43	22	1120	3500
44	Sample 44	22	1120	1300
45	Sample 45	25	1110	1800
46	Sample 46	25	1110	600
47	Sample 47	25	1110	1200
48	Sample 48	25	1110	1000
49	Sample 49	22	1180	1300
50	Sample 50	22	1180	2100
51	Sample 51	22	1180	1500
52	Sample 52	22	1180	1400

53	Sample 53	27	1190	2300
54	Sample 54	27	1190	2500
55	Sample 55	27	1190	2200
56	Sample 56	27	1190	2500
57	Sample 57	22	1190	1600
58	Sample 58	22	1190	1200
59	Sample 59	22	1190	2100
60	Sample 60	22	1190	1800
61	Sample 61	24	1170	1600
62	Sample 62	24	1170	900
63	Sample 63	24	1170	1800
64	Sample 64	24	1170	1400
65	Sample 65	16	1150	1600
66	Sample 66	16	1150	1200
67	Sample 67	16	1150	1600
68	Sample 68	16	1150	1100
69	Sample 69	33	1160	1300
70	Sample 70	33	1160	1400
71	Sample 71	33	1160	1500
72	Sample 72	33	1160	1600
73	Sample 73	21	1190	2700
74	Sample 74	21	1190	2400
75	Sample 75	21	1190	2100
76	Sample 76	21	1190	1400
77	Sample 77	22	1020	1300
78	Sample 78	22	1020	1500
79	Sample 79	22	1020	1700
80	Sample 80	22	1020	1300
81	Sample 81	26	990	2700
82	Sample 82	26	990	1100
83	Sample 83	26	990	2400
84	Sample 84	26	990	2500
85	Sample 85	33	1150	1100
86	Sample 86	33	1150	1200
87	Sample 87	33	1150	1400
88	Sample 88	33	1150	900
89	Sample 89	24	1140	1000
90	Sample 90	24	1140	1400
91	Sample 91	24	1140	1100
92	Sample 92	24	1140	1300
93	Sample 93	39	1160	900
94	Sample 94	39	1160	1200
95	Sample 95	39	1160	1100
96	Sample 96	39	1160	900
97	**Average**	25	1147	1746
98	**Max**	39	1220	3500
99	**Min**	15	990	500
